# The Impact of Breast Density Notification on Anxiety in South Australian Women Undergoing Breast Cancer Screening

**DOI:** 10.1155/ijbc/9997077

**Published:** 2025-04-25

**Authors:** Avisak Bhattacharjee, David Walsh, Pallave Dasari, Leigh J. Hodson, Suzanne Edwards, Sarah J. White, Deborah Turnbull, Wendy V. Ingman

**Affiliations:** ^1^Discipline of Surgical Specialties, Adelaide Medical School, The Queen Elizabeth Hospital, University of Adelaide, Woodville South, South Australia, Australia; ^2^Robinson Research Institute, University of Adelaide, Adelaide, South Australia, Australia; ^3^School of Public Health, University of Adelaide, Adelaide, South Australia, Australia; ^4^School of Clinical Medicine, University of New South Wales, Sydney, New South Wales, Australia; ^5^School of Psychology, University of Adelaide, Adelaide, South Australia, Australia

## Abstract

**Purpose:** The purpose was to investigate the impact of breast density notification on anxiety using the State and Trait Anxiety Inventory (STAI) tool in South Australian women undergoing breast cancer screening.

**Methods:** A survey-based cross-sectional mixed method study was conducted in women attending breast cancer screening at the Queen Elizabeth Hospital Breast/Endocrine outpatient department (*n* = 100). The women had participated in a previous study assessing their general knowledge of breast density and had indicated they wanted to know their own breast density. Breast density was assessed using Volpara software, and the participants were notified by letter. The STAI tool was administered with an additional question asking how participants felt after being told their breast density. State and trait anxiety levels were compared between those receiving notification of dense breasts and those notified of nondense breasts.

**Results:** State anxiety scores were not different between women notified they had dense breasts (*n* = 34, mean state anxiety ± SD; 36.65 ± 13.03) and those who had nondense breasts (*n* = 66, 35.17 ± 13.60, *p* = 0.51). Severe trait anxiety was observed in 8 of 34 (23%) and 13 of 66 (20%) women in the dense and nondense groups, respectively, and there were no significant differences. Qualitative analysis of 122 coded responses revealed the majority of reactions to breast density notification were positive or neutral, with 17% being negative.

**Conclusion:** Notification of dense breasts was not associated with elevated anxiety when compared to the notification of nondense breasts. Breast density notification approaches need to be considerate of the significant proportion of women with severe underlying anxiety.

## 1. Introduction

Breast density is related to the proportion of the fibroglandular tissue to fatty tissue in the breast; the dense tissue is predominantly fibroglandular tissue and appears white, and nondense fatty tissue appears dark [1]. Breast density is an independent risk factor for breast cancer and can mask cancers on a mammogram [2, 3]. While notifying women of their breast density when they have a mammogram cancer can assist in decision-making around how best to manage their breast health, there are several concerns with this practice [4]. One concern is the potential for increased anxiety and confusion among women. Studies have suggested that women notified of their breast density can report feelings of anxiety, worry, and confusion regarding their breast health [5–7].

Despite these concerns, there is a growing number of women being notified of their breast density when they have a mammogram. Breast density notification was legislated by the US Food and Drug Administration in 2023 [8]. The European Union Society of Breast Imaging also recommend informing women about their individual breast density and breast MRI for women with high breast density [9]. A number of jurisdictions in Canada also notify women of their breast density as part of community-based screening programs [10]. In Australia, the Royal Australian and New Zealand College of Radiologists updated their position statement in favour of notification in 2023 [10, 11]. BreastScreen Australia, the national umbrella organisation for breast screening does not record or report breast density to clients [11], although notification has been implemented in three different state-based BreastScreen programs [12–14].

Anxiety can be defined as “an abnormal and overwhelming sense of apprehension and fear often marked by physical signs (such as tension, sweating, and increased pulse rate), by doubt concerning the reality and nature of the threat, and by self-doubt about one's capacity to cope with it” [15]. State anxiety manifests as transient feelings of apprehension, worry, and fear elicited by a specific stimulus, while trait anxiety denotes an individual's inherent tendency towards experiencing anxiety, constituting a deeply ingrained aspect of their personality [15]. Trait anxiety is shaped by a multitude of factors including genetic predisposition, physiological mechanisms, hormonal influences, environmental context, cultural background, and societal influences [16–18]. While trait anxiety can exacerbate and intensify state anxiety, it is important to recognise that these two forms of anxiety may also possess distinct characteristics and may evolve independently of one another [17].

Women's psychological constructs and attitudes to breast density notification remain underexplored [6]. Studies that investigate anxiety associated with breast density notification within community-based public screening cohorts have typically relied on self-reported anxiety measures, not validated psychological tools [5, 19]. This is primarily due to limitations in the study setting and the possibility for the psychological tool to escalate the psychological stress of the participant.

The principal aim of this study was to investigate anxiety status and the reaction of Australian women to breast density notification using the State and Trait Anxiety Inventory (STAI) tool. The study was conducted within the setting of breast cancer screening in a large public hospital, which enabled a more probing investigation of anxiety measures compared to those conducted in the setting of community-based screening.

## 2. Methods

### 2.1. Study Design and Setting

**Figure 1:**
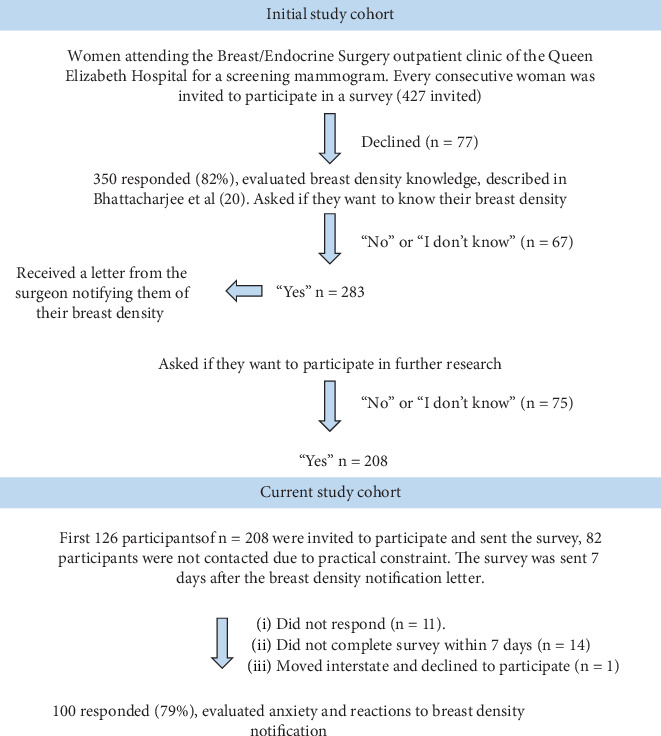
Flowchart of participation in breast density studies. The cohort for the current study was a subset of the initial cohort who had participated in a study assessing breast density knowledge.

A cross-sectional mixed methods survey was conducted between February and October, 2023 at the Breast and Endocrine public outpatient services of the Queen Elizabeth Hospital (TQEH), in Adelaide, South Australia (Figure 1).

### 2.2. Participant Recruitment and Selection

Female participants were recruited from an existing consecutive cohort who had participated in an earlier study assessing knowledge of breast density [20]. The cohort for this initial study was over 18 years of age, not suspected to have breast cancer, and attending TQEH for a routine mammogram. The participants were not part of the community-based BreastScreen Australia screening program, rather they were a hospital-based cohort attending the Breast and Endocrine outpatient department at regular intervals for routine mammograms. They did not have any breast cancer symptoms at the time of the mammogram. There are several indications for undergoing hospital-based routine mammography including previous breast cancer or benign condition, strong family history, or a genetic predisposition. Therefore, this cohort may be more concerned about their breast health in comparison to those attending community-based screening. Women were excluded if they were unable to provide informed consent and/or appeared to the researcher to be distressed. All participants provided informed consent for the study.

### 2.3. Data Collection and Procedure

The following questions were asked in the initial study: “You are waiting to have a mammogram at TQEH, and this will show your breast density. Would you like to be told your breast density?” and “The Queen Elizabeth Hospital is conducting a number of studies about breast density. If you are eligible for any studies, would you like a researcher to contact you?”

Participants who answered “yes” to the former question received the breast density notification letter by post from the consultant breast surgeon (D.W.). The letter was codesigned by the authors and a representative from the Australian Breast Density Consumer Advisory Council. It contained a breast density assessment obtained by Volpara software version 3.4 employing the BI-RADS 5th edition breast composition classification. Participants were notified their breast density was Category A, B, C, or D. The letter described the relevant breast density category using the descriptors provided by Volpara, with Category A and B being described as nondense and Category C and D being described as dense [21].

Participants who also answered “yes” to the latter question, received either an anonymised online survey (Qualtrics software) or a hard-copy survey containing the 6 items from the Mind Garden STAI that evaluates how the participant feels in the last 7 days and how they feel generally [15]. The survey was sent 7 days after the notification letter. Reminders via phone and text message were sent after 48 h of sending the survey. Participants were required to respond to the survey within the next 7 days in order to be included in the study.

The following free-text, no word limit question was also included in the survey “How do you feel after being told about your breast density?”

### 2.4. Outcomes

State and trait anxiety scores were calculated from the Mind Garden manual, with the maximum anxiety scores of both state and trait anxiety being 80 [15]. Participants were categorised as having state and trait anxiety in the following ranges: “no or low anxiety” (scores between 20 and 37), “moderate anxiety” (scores between 38 and 44), and “severe anxiety” (scores between 45 and 80) [22]. Outcomes were analysed in relation to participants' breast density status, with those receiving a letter notifying them of Category A and B being considered “nondense,” and those receiving a letter notifying them of Category C or D being considered “dense.” These categories were used based on the descriptors provided in the breast density notification letter.

### 2.5. Data Analysis

Data from the STAI tool were analysed using IBM SPSS software version 28, with missing data managed using imputation method (mode of the respective category substituted for categorical data). Categorical data and continuous data were analysed, respectively, using the chi-square test or Mann–Whitney *U* test. Effect sizes were measured using Cohen's *d* [23], and *p* values were considered statistically significant at < 0.05.

Responses to the open-ended question were analysed by an iterative 3-step summative content analysis method [24] with the researchers (A.B., D.T., and W.V.I.) blinded to breast density status of the participant. To enhance transparency and analytic rigor, the findings from the six unique themes produced by this process were reviewed by a member of the research team who is also a mammographer employed by BreastScreen Australia (L.J.H.). The responses were then unblinded and categorised according to breast density status.

### 2.6. Ethics Approval

The study was conducted in accordance with the NHMRC National Statement on the Ethical Conduct of Human Research (2007) and approved by the Human Research Ethics Committee of Central Adelaide Local Health Network (protocol #16630 approved 01 November 2022).

## 3. Results

### 3.1. Sample

A total of 427 women who were attending the Breast/Endocrine outpatients department for routine mammography were identified as eligible for the initial research and were invited to participate. Of these, 350 (81.9%) women took part in the earlier study and 208 (59%) wanted to know their breast density and wanted to be contacted about future research studies (Figure 1). Due to time constraints and practical considerations, only the first 126 were sent questionnaires, of whom 100 (79%) replied within the prespecified period of 7 days after being sent the survey. Of these, 34 women had been notified they had dense breasts and 66 had been notified they had nondense breasts (Table 1). Women had a mean age of about 60 years. All participants identified as speaking English at home. Nearly half of the participants were from the most disadvantaged areas in terms of the SEIFA and two-thirds had had three or more mammograms in the last 3 years.

### 3.2. State and Trait Anxiety

We found no statistically significant or clinically relevant differences in average state and trait anxiety scores of women in the dense and nondense breast groups (Table 2). Average scores were in the vicinity of no or low anxiety and Cohen's *d* indicated negligible to small differences between the dense and nondense groups. There were no statistically significant differences in the two groups in categorical scores for both state and trait anxiety. About a half of women in the dense breast group and 61% in the nondense group scored in the no or low state anxiety range, and about one-quarter of women in both groups had scores in the severe state anxiety range (Table 3). The distribution was similar for trait anxiety.

### 3.3. Perception After Receiving Breast Density Status

The summative content analysis produced 122 discrete codable statements generating 6 themes across the single open-ended question, “How do you feel after being told about your breast density?” (Table 4). The responses were on average 14 words long (range 1–107 words). The most common response was of a favourable nature indicating women felt informed, interested, or curious in women from both the dense and nondense groups (42% and 39% of responses, respectively). More responses from women in the dense breast group were unfavourable, indicating feelings of concern or disappointment, compared to the nondense group (19% and 5%, respectively). Conversely, fewer responses from women in the dense group indicated a feeling of gratitude, relief or reassurance compared to the nondense group (7% and 15%, respectively). About 10% of responses in both groups indicated that they felt ‘okay' after being told about their breast density. When coded in terms of positive, negative and neutral reactions, about half of comments from both groups were coded as positive and there were no significant differences between the dense and nondense group (Table 5). However, more reactions from women in the dense breast group were negative compared to reactions in the nondense group (26% and 13%, respectively).

## 4. Discussion

The current study provides critical insights into the psychological impact of breast density notification among Australian women attending breast cancer screening in a public hospital setting and provides a complimentary approach to other studies that assessed self-reported anxiety within community-based breast screening settings. Here, we use a validated psychological tool to assess anxiety following notification of dense breasts, using women notified of nondense breasts as a comparator group. Through assessing both state and trait anxiety, the impact of breast density notification can be considered against the backdrop of the underlying tendency of participants towards anxiety.

State anxiety after notification of dense breasts was not different to state anxiety following notification of nondense breasts, suggesting that psychological measures of anxiety are not affected by breast density notification. Although the sample size of 100 participants might be considered modest, this sample size is well-accepted within the psychology literature where previous studies that employed the STAI tool to measure anxiety after a stressful health event have used similar or smaller sample sizes [22, 25, 26]. The smaller effect size determined by Cohen's *d* between the dense and nondense groups in case of state (0.11) and trait (0.19) anxiety suggest that a larger sample size would not yield different results.

In this study, 20% of women who participated were identified as having severe trait anxiety. This finding is in agreement to a recent survey (2021–2022) that reported that 21% of Australian women had experienced an anxiety disorder in the last 12 months [27]. A substantial number of Australian women are suffering from high underlying anxiety in their day-to-day life and this has the potential to affect their response to any health information, including how they respond to being notified of their breast density, whether they have dense or nondense breasts.

Overall, 42% of this study cohort was identified as having state anxiety in the 7 days following breast density notification in the moderate or severe range. This is higher than other Australian study findings where 20% participants self-reported anxiety following breast density notification [5] or reported they were likely to feel anxious if told they had dense breasts [6, 28]. This difference is likely to be associated with how anxiety was measured. The STAI tool does not ask participants to assess their own anxiety, but rather measures the psychological manifestations of anxiety such as the extent of feelings of tension, worry, and calm. The high frequency of state anxiety, irrespective of whether the participant received a letter notifying them of dense or nondense breasts, might be attributed to the high level of underlying trait anxiety in the cohort.

Women who received breast density notification generally respond neutrally or positively, as reflected in the themes identified from the qualitative analysis of the open-ended question concerning how the women felt after being told their breast density. These results are similar to themes described in previous studies [5, 28]. Approximately half of the reactions were positive and reflected the participants feeling informed, relieved, reassured, and grateful. An additional 30% were neutral, with a common response being “Okay” or a statement that they were not affected by the information. Negative reactions occurred in around 20% of responses and included confusion about breast density information and concerns about breast cancer being missed.

It may not be surprising that women notified of dense breasts would experience negative reactions, given they are receiving the news that they are have an increased risk for breast cancer [29] and reduced sensitivity of a mammogram to detect cancer [30]. These negative reactions could be mitigated through codesign of health communication strategies with consumers, which has significant potential to increase community engagement and information uptake [31–33]. Trial of an interactive computer-animated agent codesigned with consumers that explains what women want to know about breast density suggests this approach can increase breast density knowledge and reduce self-reported anxiety [34]. Further work to improve breast density communication and mitigate negative reactions would benefit from engagement with consumer organisations such as the Australian Breast Density Consumer Advisory Council that assisted in the current study.

### 4.1. Limitations

Limitations of this study include data collection from a single hospital site and the potential for selection bias. The cohort was recruited from a prior study, who had specified their preference to be informed of their breast density and contacted for future studies, so the experience of those who did not want to know was not captured. Moreover, the participants were attending the public hospital-based breast service for routine mammographic screening or follow-up rather than attending community-based breast cancer screening. Women showing signs of distress at the time of their breast screening were excluded from the study, so their experiences of breast density notification were not captured. Furthermore, there was a lack of uptake of the survey by non-English-speaking women, which limits the generalizability of the study.

This study used women notified on nondense breasts as a comparator group, however it is possible that the notification letter could have increased state anxiety in the nondense group. Furthermore, this study did not distinguish between women's responses to being notified of Category C or Category D breast density, and it is possible that notification of Category D may have a greater psychological impact than Category C. Ideally, state anxiety could be measured before and after breast density notification; however, this was not possible in this study. The opportune time to measure state anxiety before notification would be when participants were recruited to the study, prior to the mammogram and their breast density measurement. However, breast screening may in itself cause some anxiety which would have affected the baseline measure [35]. Another approach would be to randomise women with dense breasts to receiving or not receiving breast density notification.

Investigation of how a participant feels on the day they receive the notification letter and in-person interviews seeking to understand how participants feel after breast density notification may have revealed more in-depth findings than the current study. In addition, the readability of the surgeons' letters and the level of health literacy of the participants were also not assessed in this study.

## 5. Conclusion

With more and more women being notified of their breast density when they have a mammogram, it is important to understand the psychological impact of this information. This study has used a validated psychological tool and suggests that notification of high breast density is not associated with an escalation in anxiety. While women's reactions to breast density notification are overall positive or neutral, negative reactions including confusion and concern can also occur. Breast density notification approaches need to be considerate of the significant proportion of women with severe underlying anxiety. Codesign of communication strategies with consumers may help mitigate negative reactions to breast density notification.

## Figures and Tables

**Table 1:** Sociodemographic variables of the participants.

**Variables**	**Dense breast** **(** **n** = 34**)** **n (%)**	**Nondense breast** **(** **n** = 66**)** **n (%)**	**Overall in current study** **(** **N** = 100**)**	**Overall in prior study** **(** **N** = 350** ) [**20**]**
Age (mean ± SD)	59.60 ± 10.89	61.09 ± 9.53	60.58 ± 9.98	61.10 (±11.45)
Marital status				
Married de facto	20 (59)	45 (68)	65 (65)	215 (61)
Never married	6 (18)	8 (12)	14 (14)	46 (13)
Separated/divorced	6 (18)	9 (14)	15 (15)	63 (18)
Widowed	2 (6)	4 (6)	6 (6)	26 (7)
Language spoken at home				
English	34 (100)	66 (100)	100 (100)	330 (94)
Others	0 (0)	0 (0)	0 (0)	20 (6)
SEIFA				
Most disadvantaged (lowest third of decile)	14 (41)	34 (51)	48 (48)	180 (51)
Moderately advantaged (middle third of decile)	15 (44)	22 (33)	37 (37)	111 (32)
Highly advantaged area (highest third of decile)	5 (15)	10 (15)	15 (15)	59 (17)
Mammogram in last 3 years				
1–2	9 (26)	21 (31)	30 (30)	136 (39)
≥ 3	25 (74)	45 (68)	70 (70)	214 (61)

**Table 2:** Average STAI scores according to breast density (*N* = 100).

**Anxiety types**	**Dense breast** **(** **n** = 34**)**		**Nondense breast** **(** **n** = 66**)**		**p** ** value**	**Cohen's ** **d**
	**Variability**	**95% CI**	**Variability**	**95% CI**		
State						
Mean (SD)	36.65 (13.03)	32.10, 41.20	35.17 (13.60)	31.83, 38.51	0.51	0.11
Median (IQR)	37 (22.25, 47.75)		33 (20, 47)			
Trait						
Mean (SD)	37.62 (11.44)	33.63, 41.61	35.19 (12.51)	32.11,38.27	0.26	0.19
Median (IQR)	37 (29.25, 44)		33 (26,43)			

*Note:p* value is significant at < 0.05. *p* value is determined by the Mann–Whitney *U* test.

**Table 3:** Distribution of participants based on anxiety and breast density types (*N* = 100).

**Anxiety types**	**Dense breast** **(** **n** = 34**)** **( n , %)**	**Nondense breast** **(** **n** = 66**)** **( n , %)**	**p** ** value**
State			
No or low	18 (53)	40 (61)	0.69
Moderate	6 (18)	8 (12)	
Severe	10 (29)	18 (27)	
Trait			
No or low	18 (53)	40 (60)	0.76
Moderate	8 (23)	13 (20)	
Severe	8 (23)	13 (20)	

*Note:p* value is significant at < 0.05. *p* value is determined by the chi-square test.

**Table 4:** Distribution of preliminary themes according to the density status (*N* = 122).

**Themes**	**Dense breast** **(** **n** = 43**)**^**a**^ **n (%)**	**Nondense breast** **(** **n** = 79**)**^**b**^ **n (%)**	**Overall** **(** **N** = 122**)** **n (%)**	**Example**
Favourable response/informed/interested/curious	18 (42)	31 (39)	49 (40)	“Very pleased to know my risk and the need to be vigilant”
Gratitude/feels relieved/feels reassured	3 (7)	12 (15)	15 (12)	“Its good to know my density as it puts my mind at rest.”
Unfavourable response/disappointed	8 (19)	4 (5)	12 (10)	“A bit concerned that a cancer may be missed on mammograms due to breast density----.”
Confused	3 (7)	6 (8)	9 (7)	“---with it, not quite sure I understood it all.-----”
Neutral	6 (14)	18 (23)	24 (20)	“Do not affect me at all.”
Okay	5 (11)	8 (10)	13 (11)	“Ok with it-------------------”
Total	43 (100)	79 (100)	122 (100)	

^a^Total response from 34 dense breast women is 30. The number of missing responses is 3, and one is contextual response. Total 30 complete responses were coded as 43 coded responses.

^b^Total response from 66 dense breast women is 59. The number of missing responses is 5, and two are contextual responses. Total 59 complete responses were coded as 79 coded responses.

**Table 5:** Final arrangement of themes according to the density status (*N* = 122).

**Themes**	**Dense breast** **(** **n** = 43**)** **n (%)**	**Nondense breast** **(** **n** = 79**)** **n (%)**	**p** ** value**	**Overall** **(** **N** = 122**)**
Negative reaction^a^	11 (26)	10 (13)	0.19	21 (17)
Positive reaction^b^	21 (48)	43 (54)		64 (52)
Neutral reaction^c^	11 (26)	26 (33)		37 (30)
Total	43 (100)	79 (100)		122 (100)

*Note:p* value has been determined by the chi-square test. *p* value is significant at < 0.05.

^a^Negative reaction consists “confused" and “disappointed/negative reaction” codes from Table 4.

^b^Positive reaction consists of “positive reaction/informed /interested/curious” and “gratitude/feels relieved/feels reassured” codes from Table 4.

^c^Neutral reaction consists of “neutral” and “okay” codes from Table 4.

## Data Availability

The data that support the findings of this study are available on request from the corresponding author. The data are not publicly available due to privacy or ethical restrictions.
